# Microbial communities in developmental stages of lucinid bivalves

**DOI:** 10.1038/s43705-022-00133-4

**Published:** 2022-07-08

**Authors:** Sarah Zauner, Margaret Vogel, Julia Polzin, Benedict Yuen, Marc Mußmann, El-Hacen M. El-Hacen, Jillian M. Petersen

**Affiliations:** 1 Division of Microbial Ecology, Department for Microbiology and Ecosystem Science, University of Vienna, Centre for Microbiology and Environmental Systems Science, Djerassiplatz 1, 1030 Vienna, Austria; 2 University of Vienna, Doctoral School in Microbiology and Environmental Science, Djerassiplatz 1, 1030 Vienna, Austria; 3 Conservation Ecology Group, Groningen Institute for Evolutionary Life Sciences, University of Groningen, P.O. Box 11103, 9700CC Groningen, The Netherlands; 4 Parc National du Banc d’Arguin (PNBA) Chami, B.P. 5355 Wilaya de Dakhlet Nouadhibou, Mauritania

**Keywords:** Animal physiology, Microbiome, Symbiosis

## Abstract

Bivalves from the family *Lucinidae* host sulfur-oxidizing bacterial symbionts, which are housed inside specialized gill epithelial cells and are assumed to be acquired from the environment. However, little is known about the *Lucinidae* life cycle and symbiont acquisition in the wild. Some lucinid species broadcast their gametes into the surrounding water column, however, a few have been found to externally brood their offspring by the forming gelatinous egg masses. So far, symbiont transmission has only been investigated in one species that reproduces via broadcast spawning. Here, we show that the lucinid *Loripes orbiculatus* from the West African coast forms egg masses and these are dominated by diverse members of the Alphaproteobacteria, Clostridia, and Gammaproteobacteria. The microbial communities of the egg masses were distinct from those in the environments surrounding lucinids, indicating that larvae may shape their associated microbiomes. The gill symbiont of the adults was undetectable in the developmental stages, supporting horizontal transmission of the symbiont with environmental symbiont acquisition after hatching from the egg masses. These results demonstrate that *L. orbiculatus* acquires symbionts from the environment independent of the host’s reproductive strategy (brooding or broadcast spawning) and reveal previously unknown associations with microbes during lucinid early development.

## Introduction

Marine invertebrates have a remarkable range of reproductive modes, but in general there are two major strategies: 1) broadcast spawning, where gametes are released to the water column where fertilization occurs, and 2) brooding, where early development occurs in “masses” within or close to the parent [1]. These two modes have major consequences for dispersal capacity, as broadcast larvae are usually numerous, long-lived, and planktotrophic, whereas brooding species either produce viviparous juveniles or lecithotrophic larvae which usually settle close to their parental populations [2].

Gelatinous egg mass formation as a form of external brooding is a common reproductive strategy in marine invertebrates. It confers many advantages either through chemical defense and the production of antibacterial compounds [3, 4], or through protection from solar radiation and desiccation [5]. Egg mass brooding has been observed in fish [6], crustaceans [7], echinoids [8], polychaetes [9], and trochid gastropods [10] but this strategy is rare in bivalves. External egg masses have so far only been reported for six species of marine bivalves: the protobranchs *Nucula delphinodonta* and *Turtonia minuta*, the cardiid *Parvicardium exiguum*, the semelid *Abra tenuis*, and the chemosymbiotic lucinids *Phacoides pectinatus* and *Loripes orbiculatus* [11–13].

The bivalve family *Lucinidae* is one of the most species-rich families in the ocean today [14]. Their characteristic feature is a conspicuous symbiosis with sulfur-oxidizing gammaproteobacteria, which live inside host cells called bacteriocytes in the gill epithelia [14]. In this intimate mutualistic association, the host provides the symbiont with reduced compounds such as hydrogen sulfide and oxygen, driving the symbiont primary production that supports a substantial fraction of the animal host’s nutrition [15, 16]. Lucinid bivalves are found worldwide and can be enormously abundant. Some seagrass sediments can have >4000 individuals per m^2^ [17] and, in seagrass meadows these bivalves clearly play a key role in ecosystem functioning [18–20]. Despite this, little is known about their life cycle in the wild, although they are all assumed to lack symbionts in their early life stages and acquire them from the environment during development [21]. Further, “broadcast” spawning, the release of gametes into the water column, has been induced in the laboratory for the lucinid *Codakia orbicularis* [22].

In this study, we investigated reproduction and symbiont transmission in *Loripes orbiculatus*, which is distributed along the English coast, throughout the Mediterranean, Black Sea, and Atlantic coasts, to West Africa [11, 12, 18, 23]. In Mauritania, this species spawns seasonally, with a major spawning event in winter (December–January) and a minor one in summer (July), by releasing gelatinous egg masses that get attached to seagrass blades on intertidal mudflats [24]. Our goal was to investigate the microbial communities associated with the early life stages of a lucinid species that reproduces via brooding. In particular, we asked whether the gill endosymbiont that associates with the adults can be detected in these early life stages of a brooding host species, which would contrast with the horizontal transmission mode demonstrated for other lucinid species.

## Material and methods

### Sample collection and fixation

**Fig. 1:**
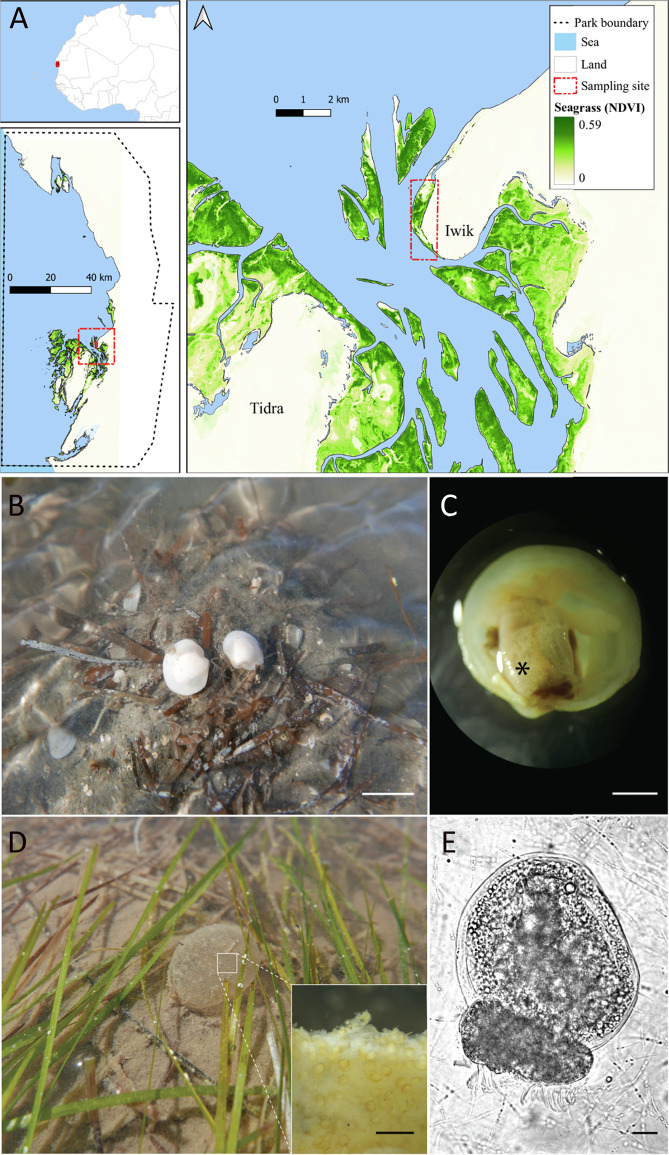
Developmental stages of *L. orbiculatus* in intertidal seagrass mudflats at Banc d’Arguin National Park, Mauritania. **A** Map showing the sample locations including seagrass densities in the study area based on the median Normalized Difference Vegetation index (NDVI) of the Sentinel-2 Images (Map created in GoogleEngine, filter date; “2020-01-01”, “2021-03-03”). **B** Adult bivalves on seagrass debris; scale bar = 1.2 cm. **C** Adult *L. orbiculatus* visualized under a stereo microscope, the asterisk marks one of the symbiont-housing organs, the gills; scale bar = 0.3 cm. **D** Egg mass (approximately 4 cm in size) of *L. orbiculatus* attached to seagrass leaves. Larvae are embedded in a gelatinous, collagen-like fiber structure; scale bare = 200 µm. **E** Veliger larvae inside the egg mass; scale bar = 20 µm.

Egg masses and adult bivalves were collected in two consecutive seasons (December 2015 and January 2017) from intertidal seagrass mudflats around the village of Iwik located in the Banc d’Arguin National Park, Mauritania, West Africa (19°53.82′N, 16°18.63′W; Fig. 1). Developmental stages and adult specimens for molecular analyses were rinsed with 0.2 µm sterile, filtered seawater, and immediately fixed in RNAlater (Cat. No. AM7020; Life Technologies, Carlsbad, CA, USA) at 4 °C overnight and stored at −20 °C until extraction. Three adult *Loripes orbiculatus* individuals were dissected and gills were fixed in 4% paraformaldehyde (PFA), dehydrated into 70% ethanol, and stored at 4 °C until further analysis (Supplementary Methods). Sediment and seagrass rhizome debris was sampled in December 2015 for microbial community analysis. Samples (*n* = 5) were collected at the site using aseptic techniques from 0 to 8 cm depth, fixed in 96% ethanol and stored at −20 °C until further processing.

Live egg masses were brought back to the laboratory for hatching and kept individually in petri dishes (100 mm diameter) provided with native, sterile sand and 0.2 µm sterile, filtered seawater (salinity 40‰) without any additives. The water was exchanged daily, and the development of larvae was monitored over a course of 54 days. Three larvae from each of the four sampling points (prior to hatching, 10, 12, and 54 days post hatching) were fixed for histological analysis in 4% PFA (in 0.01 M PBS, 10% sucrose), washed, dehydrated, and stored at 4 °C until embedding.

### Histological sample processing

Adult gill tissue was embedded according to [25] with modifications (Supplementary Methods). A Leica RM2235 microtome (Leica, Nussloch, Germany) was used to cut the embedded gills into 5 µm sections. Dewaxing was performed with 90%, 80%, 70%, and 50% ethanol for 5 min each. Larvae were embedded in LR-White low viscosity resin (London Resin Company, London, UK) as described in ref. [26] and cut into 1-µm semithin sections using a Leica EM UC7 Ultramicrotome. For histological evaluation toluidine blue was applied to all sections, which were evaluated on an AxioImager A1 microscope (Zeiss, Oberkochen, Germany).

### Fluorescence in situ hybridization (FISH)

Twelve larvae (three individuals from each of the four-time points) and gill tissues from three adult bivalves were hybridized with a symbiont-specific and general bacterial probes (Table S2; refs. [27–29]) in a 35% formamide buffer. The nonsense probe NON-388EUB was used to detect nonspecific binding. Slides with reaction mixture were incubated at 46 °C for 3 h in dark conditions (Supplementary Methods, Table S3). After hybridization, the slides were washed with pre-warmed (48 °C) washing buffer for 15 min and dried with compressed air. The sections were stained with 10 μg/ml DAPI and incubated in the dark for 5 min. After staining, slides were washed in ice-cold MilliQ water, mounted with Citifluor^TM^ antifade mounting medium (Citifluor Products, Canterbury, UK) and stored at 4 °C. Sections were evaluated on a TCS SP8 X confocal laser scanning microscope (Leica, Wetzlar, Germany).

### DNA extraction and marker gene amplification

DNA from gill tissues of four adult bivalves and from small pieces of 26 egg masses containing larvae was extracted using the DNAeasy Blood & Tissue Micro Kit (Qiagen, Hilden, Germany) following manufacturer’s instructions. Seven additional egg mass samples, from which larvae had been manually removed under aseptic conditions prior to DNA extraction served as controls. DNA from sediment and seagrass rhizome debris was extracted with the PowerSoil^®^ DNA Isolation Kit (MoBio Laboratories, Inc., Carlsbad, CA, USA) following manufacturer’s instructions. The purified DNA samples were quantified using the Qubit 2.0 broad range assay (Life Technologies).

For molecular characterization of the host, we used the mitochondrial cytochrome b gene for PCR-based screenings. The following primers were used to amplify a 355 bp fragment of the lucinid *cytB* gene: cytb-F [30] and cytbR_new [31]. For DNA barcoding of the symbiont, the universal bacterial primers 616V and 1492R [32, 33] were used to amplify an ~1400 bp fragment of the 16S rRNA gene. The primer sequences, amplification protocols, and cycling conditions used for *cytB* and 16S rRNA gene PCRs are in Tables S2 and S4. Purified DNA (ZR96 DNA Cleanup Kit; Zymo Research, Orange, CA, USA) was sent for Sanger sequencing at Microsynth Austria.

### 16S rRNA amplicon sequencing

DNA from the egg masses, sediment, and seagrass rhizome debris was amplified in a two-step barcoding approach as described in [34] using the primer pair 341F/785R [35]. The first step PCR was done in triplicates for all samples to minimize stochastic PCR biases. Primers and detailed PCR protocols including cycling conditions are given in the Supplementary Material (Tables S1, S3). The triplicate PCR products were pooled and the ZR96 DNA Cleanup Kit (Zymo Research) was used, according to the manufacturer’s protocol, to purify the pooled PCR products to avoid carryover of primers and primer dimers to the second-step PCR.

Second step PCR reactions consisted of the same reagents as in the first step PCRs with one amendment—each reaction contained 50 µM barcode primers per reaction (Table S4). The second amplification step for egg mass PCR products was performed under the same conditions as described for the first step, but in 10 cycles, to avoid amplification biases due to high cycle numbers. The sediment and seagrass rhizome debris cycling conditions are provided in Table S4. The second step PCR products were purified using the same procedure as the first step PCR products. Purified PCR products were quantified using the Quant-iT^TM^ PicoGreen^®^ dsDNA Assay Kit (Invitrogen Life Technologies, Gaithersburg, MD, USA). All samples were pooled together in equimolar amounts and sent for Illumina MiSeq (2 × 250 bp) sequencing at Microsynth Austria.

### Sequence processing and statistical analysis

Raw sequences were demultiplexed following the methods described in ref. [34] and further processed using dada2 v. 1.14.1 [36] in R v. 3.6.1 [37]. The demultiplexed reads were quality filtered, after which a total of 38 samples remained (26 from egg masses, seven from egg masses without larvae, and five from sediment and rhizome debris).

The reads from these samples were joined and an amplicon sequence variant (ASV) table was constructed. The ASV table was subsequently filtered to remove chimeras as well as any sequences resulting from mitochondrial or chloroplast DNA and taxonomy was assigned using the SILVA database v. 138.1 [38–40]. Singletons and doubletons were removed. For the detection of potential cross-contamination, we identified taxa that had higher total counts in any other sample library run on the same plate excluding the sample library analyzed in this study [34, 41]. These flagged taxa were then removed from the egg mass and sediment data when observed only in the contaminated samples from one sequencing run.

Additional 16S rRNA amplicon sequence datasets were obtained from NCBI’s SRA database (BioProject PRJNA282077 and PRJNA41903; from refs. [42, 43], respectively) to compare egg mass microbial community composition with the microbiota commonly found in the surrounding seagrass habitat. As 16S rRNA amplicon data from living seagrass at the sampling site was not available, we used amplicon sequences from seagrasses belonging to the genera *Zostera* and *Cymodocea*, which also occur in Mauritania. Specifically, external datasets contained 16S rRNA amplicon sequences from *Zostera japonica* (Netarts Bay, Oregon, United States), *Zostera marina* (Netarts Bay, Oregon, United States and Culatra Island, Portugal), *Zostera noltei* (Culatra Island, Portugal), and *Cymodocea nodosa* (Culatra Island, Portugal), as well as amplicon sequences from seagrass associated sediments and overlying seawater (Culatra Island, Portugal; Table S1). These sequence data were processed separately following the same protocol as previously described. The resulting ASV tables from all datasets were then merged using taxonomic assignments and the resulting table was normalized to account for compositional bias and differences in sequencing depth among samples with the Wrench package [44] using the *W*_*2*_ estimator.

All statistical analyses were conducted on the normalized ASV table using R v. 3.6.1 (ref. [37]). Diversity metrics, including Shannon–Wiener Diversity (*H’*) and Pielou’s evenness index (*J*), were calculated using the vegan package [45]. The distribution and homogeneity of variance in the data were determined using the Shapiro-Wilk Test and Levene’s Test to select the appropriate parametric (Student’s two-sided *t*-test) and nonparametric tests (Wilcoxon and Kruskal-Wallis Rank Sum). Differences in microbial community composition (normalized taxonomic group abundance and identity) were examined using non-metric multidimensional scaling ordination with Bray–Curtis distance in two dimensions. Community compositional differences due to sample type were assessed using permutational multivariate ANOVA (PERMANOVA) with the vegan package [45]. To identify taxonomic groups that had a significant association with egg masses, indicator species analysis was conducted using a point biserial correlation coefficient with the indicspecies package [46, 47] and resulting *p* values were adjusted using Benjamini–Hochberg’s False Discovery Rate (FDR correction).

### Phylogenetic analysis of host and symbiont marker genes

For phylogenetic reconstruction of the host mitochondrial cytochrome B gene, 17 full-length sequences were extracted from lucinid metagenomes from the NCBI SRA database (BioProject PRJNA679177 from [48]). We assembled mitochondrial genomes from the metagenome read libraries to obtain peptide sequences of the cytochrome B gene, which has been established as a marker gene in lucinid molecular taxonomic studies [49]. Adapter-trimmed reads were used as input for mitochondrial genome assembly with Novoplasty on default parameters and using a publicly available *L. orbiculatus* mitochondrial genome (EF043341.1) as the seed sequence [50]. The mitochondrial genome assemblies were then functionally annotated using the MITOS2 webserver [51].

In addition to the metagenome-obtained sequences, we used two full-length *cytB* gene sequences from GenBank to generate a matrix (accession no. EF043341.1 and EF043342.1). In total, sequences of 15 *Loripes* individuals together with sequences of four individuals from three different lucinid genera: *Lucinella, Clathrolucina,* and *Divalinga* were used to generate a phylogenetic tree. The *cytB* sequence of *Divalinga quadrisulcata* was used as an outgroup. All metagenome-derived *cytB* sequences were translated into protein sequences in the software Geneious v. 11.0.3 [52] using the invertebrate mitochondrial translation table 5.

The symbiont 16S rRNA phylogeny was reconstructed using 26 near full-length sequences (>1300 bp), two of which originated from the aforementioned Bioproject PRJNA679177 while the remaining 24 sequences were obtained from GenBank (see results, Fig. S2). *Allochromatium vinosum* was used as the outgroup.

Symbiont sequences were aligned individually using MAFFT v7 [53] with the Q-INS-I algorithm [54] in Geneious. Host sequences were translated into AA sequences and aligned in MAFFT v7 with the E-INS-I algorithm. Host and symbiont phylogenies were reconstructed with the maximum likelihood-based tool IQTree [55] using the Ultrafast Bootstrap Approximation UFBoot [56] with 1000 bootstrap runs. In addition to maximum likelihood, support values were generated using approximate Bayes and SH-aLRT analyses [57, 58]. The TIM + F + I (for Maximum likelihood) and the TN + F + G4 (for Bayesian) substitution models were the best fit for the 16S alignment and the mtZOA+I (for Maximum likelihood) and mtZOA (for Bayesian) for the *cytB* alignment. Phylogenetic trees were visualized in iTol [59].

## Results

### Lucinids from Mauritania reproduce by releasing gelatinous egg masses

The early developmental stages of *L. orbiculatus*, encapsulated within gelatinous egg masses, were abundant and easily obtained during the low tide on the intertidal mudflats in Banc d’Arguin during the entire research stay (2 weeks in December 2015 and January 2017; Fig. 1). We observed that adult bivalves deposited transparent gelatinous egg masses, which were 3–4 cm in diameter and still connected to the parent’s mucus tube, by attaching them to the leaves of the seagrasses *Zostera* sp. and *Cymodocea* sp. (Fig. 1 and other personal observations). The matrix of the egg masses was characterized by a fibrous structure, which was made more conspicuous following fixation and likely comprises collagenous structural proteins such as was reported in the egg capsules of elasmobranchs [60] or the antimicrobial mucus of some heterobranch gastropods [61, 62]. The live egg masses were densely packed with trochophore and/or veliger larvae (Fig. 1). The larvae actively rotated and extended their velum within the egg masses, activities that became even more apparent after hatching.

**Fig. 2:**
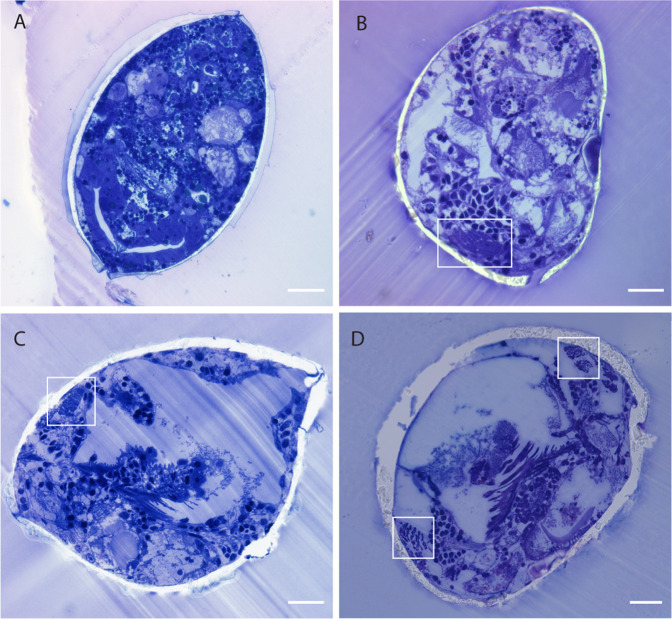
Development of *L. orbiculatus* prior to and post hatching from the egg masses. Scale bars = 20 µm. **A** Individual larva prior to hatching removed from inside the egg mass and fixed. No gills are visible. **B** Larva fixed 10 days after hatching. Individual, small gill filaments are already detectable (highlighted in white box). **C** Larva 21 days after hatching, gills visible. **D** Larva 54 days after hatching from the egg mass. Gills are shown in white boxes for **C** and **D**.

“Hatched” pediveliger larvae that had left the egg mass were observed over a period of 54 days, during which they grew from ~200 µm to ~360 µm. Fully formed gill filaments were only seen in “hatched” individuals and never in larvae while still inside the egg mass (Fig. 2). No additional gill filaments were formed, nor did the filaments appear to grow over the course of 54 days in the laboratory (Fig. 2). The absence of gill filaments in “unhatched” larvae suggest that gill development is initiated only upon hatching.

**Fig. 3:**
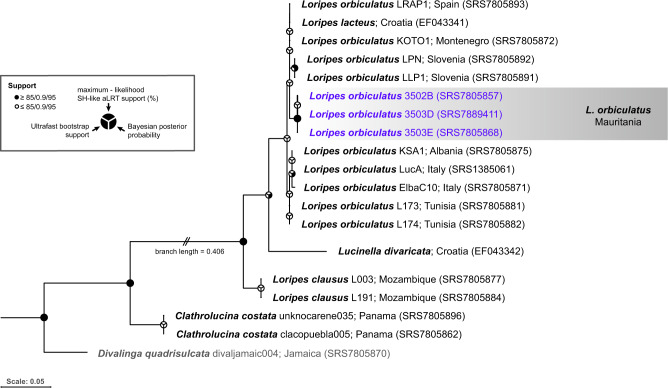
Single-gene tree for *Lucinidae* based on the mitochondrial cytochrome b gene showing that *L. orbiculatus* from Mauritania forms a distinct, monophyletic group. Support values are given in Bayesian Posterior Probability, Ultrafast Bootstrap and Maximum Likelihood SH-like aLRT; values ≥85%/0.9/95% are marked with black circle segments. Bivalves from Mauritania are highlighted in dark purple. The publicly available sequence of *Divalinga quadrisulcata* (SRS7805870) was used as the outgroup and the tree was visualized using IQ Tree.

The *cytB* sequences amplified from adult gills and egg masses were 98% identical to NCBI database entries for *Loripes lacteus*, a synonym for *Loripes orbiculatus* Poli [63] from Croatia. Phylogenetic reconstruction placed the metagenome-derived full-length *cytB* gene sequences from Mauritanian lucinids into a monophyletic, well-supported clade (95% bootstrap support) within the genus *Loripes*, although they were distinct from others sampled in Europe (Fig. 3). The closest relatives of the Mauritanian clams are those from Slovenia, Croatia, Montenegro, Spain and France. These findings suggest that there are genetic differences amongst different Mediterranean and Atlantic populations of *Loripes orbiculatus*, but further taxonomic analyses would be required to describe these differences in detail.

### Mauritanian lucinid symbionts belong to the species group *Ca*. Thiodiazotropha taylori

All of the 16S rRNA gene sequences amplified from the gills of adult *Loripes* from Mauritania were virtually identical (with single nucleotide changes) and belonged to the recently described lucinid symbiont species *Candidatus* Thiodiazotropha taylori [48]. The Mauritanian clam symbionts formed a highly supported monophyletic clade together with other described *Ca*. Thiodiazotropha in phylogenetic analysis of partial 16S rRNA genes (bootstrap support value = 94%). Like the hosts, and consistent with previous phylogenomic analyses, the closest relatives of this symbiont group were symbionts associated with the Mediterranean *L. orbiculatus* (ref. [48]; Fig. S1).

### Diverse microbes were associated with the egg masses but the intracellular symbiont was not detected

A total of 479 ASVs belonging to 174 unique taxonomic groups were common to all egg mass samples. A similar number of taxonomic groups were found in both the microbial communities from egg mass pieces including larvae (161 groups) and egg mass pieces with the larvae removed (155 groups), indicating that these organisms possibly reside in the gelatinous mass rather than on the surface of or within the larvae. Microbial community composition, including taxonomic identity and relative abundance, did not differ significantly between egg mass pieces with and without larvae (PERMANOVA, *R*^2^ = 0.02, *p* > 0.05). Additionally, median diversity (*H’*) and evenness (*J*) were not significantly different (*p* = 0.35 and *p* = 0.50, respectively) between communities from egg masses with larvae present (*H’* = 2.94; *J* = 0.90) and without (*H’* = 3.21; *J* = 0.90). As no significant differences between the two sample types were observed, the egg mass samples with and without larvae were combined for further analyses.

**Fig. 4:**
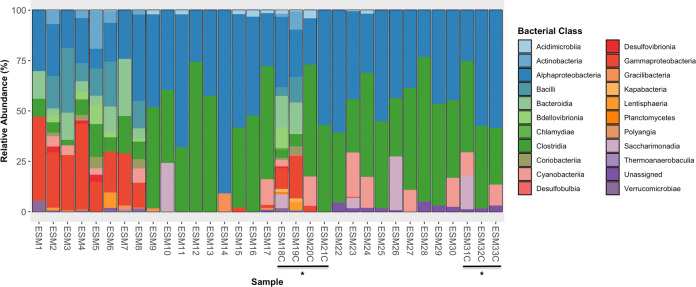
Taxonomic diversity of egg mass-associated bacterial communities based on 16S rRNA gene amplicon sequences grouped by major taxa and classified using ASVs. ESM1–ESM21C were sampled in December 2015 whereas ESM22 – ESM33C were sampled in January 2017. Asterisks mark egg mass samples with larvae removed.

Sequence reads from all egg mass samples were assigned to 21 taxonomic classes belonging to 12 phyla. The bacterial class Alphaproteobacteria (39.4%) comprised the largest portion of relative read abundance on average across all samples, followed by Clostridia (33.0%), Gammaproteobacteria (7.6%), Bacilli (3.7%), Bacteroidia (3.5%), Cyanobacteriia (4.1%), Saccharimonadia (2.4%), and Actinobacteria (1.5%; Fig. 4).

**Fig. 5:**
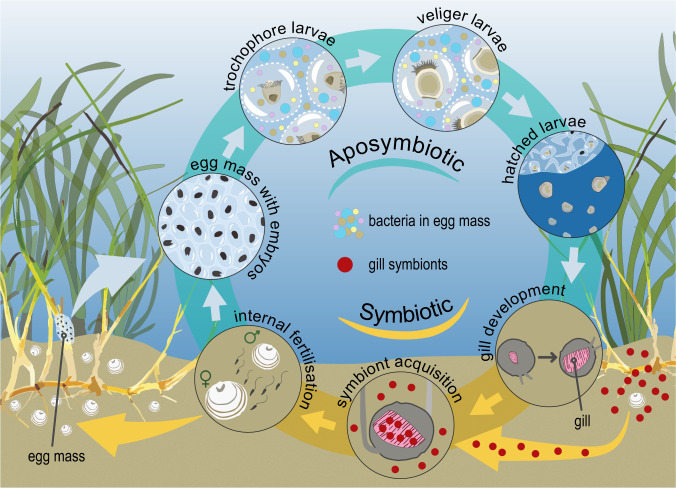
Schematic figure illustrating the life cycle of Mauritanian lucinid bivalves associated with horizontally transmitted symbionts. Egg masses are deposited by attaching them to seagrass blades while they are still connected to the mother’s mucus tube. The larvae inside the egg masses are aposymbiotic and undergo development via a trochophore (ref. [11]; ~8 days after egg mass release) and a veliger larval stage (ref. [11]; ~12 days after egg mass formation) before they hatch as mature pediveliger larvae. Gill development is initiated upon hatching and the sulfur-oxidizing endosymbionts colonize the settled juvenile. Environmental bacteria are shown in pink, blue, yellow, and brown. The specific sulfur-oxidizing symbiont is depicted in red. Broken lines indicate that we could not verify the presence of a membrane that separates the larvae inside the egg mass although such a barrier has been reported from other lucinid larvae [13]. Not shown to scale.

The remaining bacterial classes comprised less than 1% of relative read abundance. No ASVs belonging to the archaeal domain were detected in the egg mass microbial communities. The alphaproteobacterial ASVs assigned to the families *Rhodobacteraceae* and *Rhizobiaceae* comprised the largest proportions of the reads. Whereas the most abundant representatives from the Clostridia class belonged to the families *Ruminococcaceae* and *Lachnospiraceae*. None of the reads from egg mass samples were assigned to the genus *Ca*. Thiodiazotropha, which is consistent with the horizontal transmission of symbionts occurring at a later host life-stage (Fig. 5).

### Microbial communities in the egg masses are distinct from those in other seagrass microhabitats

**Fig. 6:**
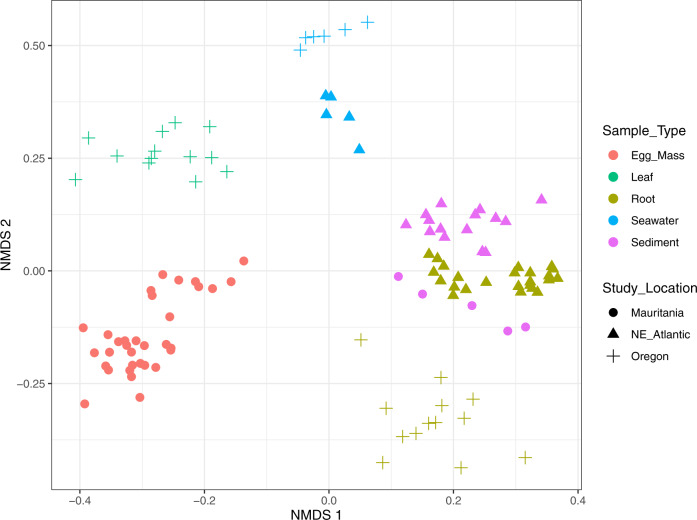
NMDS ordination plot based on 16S rRNA gene amplicon sequencing showing differential clustering of egg mass bacterial communities compared to seagrass leaves, seagrass roots, seawater and sediment. Sediment samples include material from three different depths: surface layer = 0–2 cm; intermediate layer = 2–5 cm; bottom layer = 5–8 cm. Seagrass rhizome debris sampled from the bottom layer was sieved and treated as a sediment sample. Analysis is based on data from Illumina sequencing and Bray–Curtis dissimilarities. Different study locations are given in different shapes whereas sample types are indicated by color (stress = 0.1796; *k* = 2).

As seawater, seagrass leaves and rhizome samples from Mauritania were not available, we used 16S rRNA gene amplicon sequence data from other published studies to compare the microbial communities found in *L. orbiculatus* egg masses to microbial communities typically found in the different microhabitats in a seagrass meadow (seagrass leaves and rhizomes, sediment, and seawater; Table S1). There were 838 taxonomic groups identified in all samples from the seagrass microhabitats and egg masses (*n* = 114). Microbial community composition, including both taxonomic identity and abundance, was significantly different depending on microhabitat type (PERMANOVA, *R*^2^ = 0.25, *p* = 0.001; Fig. 6; Table S5). Alpha diversity was also significantly different between the habitat types; the sediment and seagrass root microbial communities had significantly higher diversity (*H’*) than the egg mass communities (Kruskal-Wallis, *Χ*^2^ = 62.19, *p* < 0.001; Dunn’s test, adjusted *p* < 0.05; Table S6). Sediment communities had the highest diversity of any habitat type. Sediment also had the highest number of taxonomic groups, and both sediment and seagrass root microbial communities contained more taxonomic groups than the egg mass communities (Kruskal-Wallis, *Χ*^2^ = 61.477, p < 0.001; Dunn’s test, adjusted *p* < 0.05; Table S6). However, the egg mass microbial communities had significantly higher evenness (*J’*) than both the seagrass root, seagrass leaf and sediment microbial communities (Kruskal-Wallis, *Χ*^2^ = 25.999, p = 0.001; Dunn’s test, adjusted *p* < 0.05; Table S6).

While *Ca*. Thiodiazotropha was not detected in the egg mass microbial communities, sequences assigned to this genus were observed in the sediment microbial communities from Banc d’Arguin, Mauritania (*n* = 4) as well as in the seagrass rhizome (*n* = 19) and seawater communities (*n* = 1) from other locations. ASVs belonging to this genus comprised 0.88% of relative read abundance on average, ranging from 0.03 to 9.60% relative abundance in a single sample.

Indicator species analysis identified 51 taxonomic groups that showed a significant association with egg masses (point biserial correlation, adjusted *p* < 0.05) when compared with the seagrass environment. These 51 indicator taxa belonged to six bacterial phyla (Actinobacteriota, Bacteroidota, Cyanobacteria, Firmicutes, Patescibacteria, and Proteobacteria). The alphaproteobacterial genera *Oceanicaulis* and *Tropicimonas* had the strongest association with the egg mass environment. Many of the other indicator taxa also belonged to the class Alphaproteobacteria including eight that belonged to the Rhodobacterales order. The bacterial order Lachnospirales within the Firmicutes phylum was also frequently represented with 12 indicator taxa assigned to this taxonomic designation. Many of the indicator taxa we identified comprised the largest portions of relative abundance on average, including the genera *Tuzzerella* (3.29%) and *Subdoligranulum* (3.00%), which both belong to the Firmicutes phylum.

## Discussion

In Banc d’Arguin, Mauritania, *L. orbiculatus* produces gelatinous egg masses, an intriguing phenomenon considering that bivalves rarely reproduce via “external” brooding. This provides further evidence of the diversity of reproductive strategies within the *Lucinidae* family [13, 22]. Previously, one spawning and one brooding species were reported, we now confirm earlier morphological reports that the species *Loripes orbiculatus* is capable of brooding via external gelatinous egg masses [11, 12, 22, 13]. Ovipositioning in sand-dwelling molluscs such as bivalves usually requires a suitable substrate to which the animals can attach their egg capsules or masses. In soft-bottom habitats such as intertidal mudflats however, the animals need to find alternatives to rocky substrates. Some animals have overcome these limitations by traveling long distances to find suitable places for oviposition while others deposit their egg capsules onto living organisms such as algae or conspecifics [64–66]. While reports of lucinid ovipositioning in nature are scarce [67], we observed that *L. orbiculatus* egg masses were mostly attached to seagrass leaves or the leaf stem. This suggests that in a habitat lacking adequate substrates for deposition it can be advantageous to attach the egg masses to alternative substrates such as seagrass leaves to enhance offspring survival. Mutualistic interactions between bivalves and seagrasses have been hypothesized based on metabolic interactions, but a reproductive benefit for lucinids of associating with seagrasses has not been previously considered [18, 68]. This discovery may explain the recent increase in the *L. orbiculatus* population as a result of a tremendous increase in seagrass cover in the study area [69].

Observing animal reproduction in nature remains challenging and we cannot exclude the possibility that different individuals belonging to the same bivalve species may be capable of both broadcast spawning and brooding, depending on external factors such as environmental conditions. In fact, the coexistence of different modes of reproduction within closely related, co-occurring marine invertebrates is common and well-documented from asteroids [70, 71], bivalves [72], pteropods [73], ophiuroids [74, 75], chitons [76], limpets [77, 78], and nereids [79]. It was even reported that both brooding and broadcasting exist within the same female individual of the sea star species *Pteraster militaris* [80]. Although the selection pressures driving the evolution of broadcast spawning versus brooding in *Lucinidae* are unclear, we would expect the mode of reproduction to have numerous consequences for the host’s biology. For example, broadcast spawners presumably disperse greater distances than brooders [1]. This may be detectable in the genetic population structure of the animal hosts, as brooders would be expected to show lower levels of population connectivity and genetic diversity compared to broadcast spawning species as was observed in closely related species of the brittle star *Ophioderma longicauda* [75].

Another consequence of egg masses is that they provide a new habitat for other microbes. We show that diverse microorganisms were associated with the egg masses, which form a microhabitat that was distinct from other seagrass environments. There were no significant differences in egg mass bacterial community compositions over time (December 2015 and January 2017), indicating that the population structure remained stable over a period of at least 1 year. This further suggests that certain conditions or factors intrinsic to the egg masses shape community composition.

Similar bacterial communities were found before and after larvae were removed from the egg masses which implies that microbes most likely associate with the gelatinous mass rather than the larvae. We observed an additional membrane or barrier around fertilized eggs similar to the capsules surrounding larvae in *Phacoides pectinatus* [13], but individual membranes were not visible at any later developmental stage (data not shown). These observations raise the possibility that larvae were in direct contact with bacteria in the gelatinous mass from an early developmental stage. Imaging studies such as FISH in the egg masses would be needed to pinpoint the exact location of the bacteria.

Phytoplankton blooms that preceed *L. orbiculatus* spawning events in May and between November and December [81, 24] might explain the abundance of cyanobacteria (predominantly from the order Phormidesmiales and Synechococcales) and diatoms in the egg masses. Some members of these orders supply oxygen to larvae in egg masses of various aquatic invertebrates and amphibians and are therefore thought to contribute to larval fitness [82–84]. Moreover, diatoms associate ubiquitously with alphaproteobacteria in many aquatic habitats [85, 86] and thus could play an important role for *L. orbiculatus* in the larval stage dominated by alphaproteobacterial groups. In addition to oxygen production, phototrophic microbes in the egg masses could provide an additional source of nutrition to the developing larvae [83, 87]. Reads belonging to the bacterial class Clostridia were surprisingly among the most abundant in the egg masses. Most of these representatives (e.g., *Ruminococcaceae, Lachnospiraceae*) are among the most abundant taxa in the gut microbiome of humans and other animals including birds [88]. Several migratory shorebird species (e.g., *Calidris pusilla*, *C. alpina*, *C. canutus*, *Arenaria interpres* and others) harbor Clostridia in their gastrointestinal tract from an early age on, which are thought to be acquired from the environment [89, 90]. The exact role of Clostridia in wild birds is still unknown but their occurrence is positively correlated with weight gain [91] and might influence the survival rates of migratory birds. The occurrence of Clostridia in egg masses could possibly be related to the presence of high numbers of these foraging shorebirds on the intertidal mudflat [92]. Understanding the functions of the egg mass-associated microbes will require additional studies; however, the known metabolic capabilities of microbes related to members of the egg mass community could shed light upon their successful colonization of the egg masses or help generate hypotheses about their potential functions (for more details see the Supplementary Discussion).

Although the egg masses host such diverse microbial communities, one particular microbe was conspicuous by its absence—the chemosynthetic symbiont that reaches extremely high abundance in all adult *L. orbiculatus*. This supports previous reports of horizontal transmission in another lucinid species, *Codakia orbicularis*, with environmental symbiont acquisition in a later life-stage of the host [22, 93]. Bacteria affiliated with the genus *Ca*. Thiodiazotropha are widespread in seagrass root environments; however, very few studies have directly compared the diversity of host-associated and free-living *Ca*. Thiodiazotropha at the same site [42, 43, 29]. We found exact matches between host-associated and free-living stages in sediment samples from Banc d’Arguin. These observations are all consistent with the acquisition of symbionts from the environment during development, independent of reproductive strategy (broadcast or brooding).

## Conclusions

This study revealed differences in the reproductive biology and molecular identity of lucinid species that were previously assigned to a single species. The genetic diversity within the genus *Loripes* is therefore probably still underestimated. Moreover, we show that differences in the life cycle of lucinids and their mode of reproduction are not necessarily linked to differences in symbiont transmission mode, which is similar to patterns observed in corals where there is no strong link between reproductive mode of the host, and transmission mode of the symbionts [94]. Future studies will reveal the ecological drivers of these different reproductive modes, and their consequences for fundamental aspects of host biology such as dispersal and population structure. In addition, if the communities of specific microbes associated with the egg masses play beneficial roles for the host, this would indicate shifts in reliance on different microbes during ontogeny, as has been observed for animals ranging from insects and echinoderms to humans (e.g., [95–97]). Finally, our observation that *Loripes* egg masses attach to seagrasses adds important components in understanding the functioning of intertidal flats and the interplay between lucinids and seagrass beds.

## Supplementary information

Supplementary Information

Table S1

Table S4

## Data Availability

The 16S rRNA gene amplicon datasets generated in this study are available in the NCBI Sequence Read Archive under BioProject accession number PRJNA783529.
