# Supplementary material for: Microbial communities in developmental stages of lucinid bivalves
Source: ISME Commun. 2022 Jul 8;2:56. doi: 10.1038/s43705-022-00133-4 (PMC9723593; doi:10.1038/s43705-022-00133-4)
Supplement: Supplementary file 1 — Supplementary Information [file 43705_2022_133_MOESM1_ESM.pdf]

Supplementary Information for
Microbial communities in developmental stages of lucinid bivalves

Sarah Zauner[1,2], Margaret Vogel[1], Julia Polzin[1], Benedict Yuen[1], Marc Mussmann[1], El-Hacen M. El-Hacen[3,4], Jillian M. Petersen[1]

[1]University of Vienna, Centre for Microbiology and Environmental Systems Science, Department for Microbiology and Ecosystem Science, Division of Microbial Ecology, Djerassiplatz 1, 1030 Vienna, Austria

[2]University of Vienna, Doctoral School in Microbiology and Environmental Science, Djerassiplatz 1, 1030 Vienna, Austria

[3]Conservation Ecology Group, Groningen Institute for Evolutionary Life Sciences, University of Groningen, P.O. Box 11103, 9700 CC Groningen, The Netherlands

[4]Parc National du Banc d'Arguin (PNBA), Chami, Wilaya de Dakhlet Nouadhibou, B.P. 5355, R.I. de Mauritanie

Corresponding author: Sarah Zauner sarah.zauner@univie.ac.at, Jillian M Petersen jillian.petersen@univie.ac.at

This PDF includes:

Supplementary text
Figures S1 and S2
Tables S1 to S6
SI References

Supplementary Information Text

SI Methods

Sample collection & preparation

For FISH, dissected adult gills and larvae were fixed in a 4% paraformaldehyde, 0.01 M PBS (pH 7.4) containing 10% (wt/vol) sucrose at 4°C for 12 hours. The gills were then washed in 10% sucrose, pH 7.4, 0.01 M PBS solution for 10 minutes, three times and dehydrated in an increasing ethanol series of 30%, 50%, and 70% for 10 minutes each. Samples were stored in 70% ethanol at 4°C until embedding.

Histological sample processing

Adult *L. orbiculatus* individual gills were embedded as follows: 1-hexadecanol was mixed with polyethylene glycol distearate 1:9, melted at 60°C and stored at 37°C. Samples were sequentially dehydrated in 70%, 80% and 96% ethanol for 30 min each, than infiltrated with 3:1, 2:1, 1:1 ethanol:wax and finally three times in pure wax for 60 min at 37°C. Embedded material was left to solidify overnight at room temperature before it was stored at -20°C. A Leica RM2235 microtome was used to cut the embedded gills into 5 µm sections which were then mounted on SuperfrostPlus adhesion slides (Thermo Scientific) in a 4°C water bath. Slides were dewaxed in a decreasing ethanol series of 90%, 80%, 70% and 50% for 5 min each. Dewaxed slides were dried with compressed air and stored at -20°C until further use.

Larvae were embedded in LR-White low viscosity resin (London Resin Company), trimmed and cut into 1-µm semithin sections using a Leica EM UC7 Ultramicrotome. Sections were subsequently mounted on SuperfrostPlus adhesion slides (Thermo

Scientific) at room temperature. Toluidine blue was applied to all sections which were evaluated on a Zeiss Axio Imager A1 microscope.

Fluorescence-in-situ-hybridisation (FISH) on larvae and adult bivalves

Adult gill sections as well as whole juvenile sections were hybridized with general bacterial probes and a symbiont-specific probe (Table S2) in a hybridization buffer containing 35% Formamide and 0.01% SDS (v/v), at a final concentration of 0.9M NaCl, 0.02M Tris-HCl (Table S3). Approximately 20 µl of buffer was used per gill section. 2 µl of each probe was added to each section. Kimtech Science precision wipes (Kimberly-Clark, USA) were added which were soaked in hybridization buffer to the hybridization chamber located underneath the slides. Samples were hybridized for 3 hours at 46°C in the dark. The samples were then washed in a buffer solution for 15 min, dipped briefly in ice-cold Milli-Q water, and dried in compressed air. Slides were then stained using 20 µl (10 µg/mL) of DAPI and incubated at room temperature in the dark for 5 min. Slides were briefly rinsed in ice-cold Milli-Q water and dried using compressed air. Dried sections were mounted with Citifluor antifade mounting medium (Citifluor Ltd., UK) and stored at 4°C. Sections were evaluated with a TCS SP8 X confocal laser scanning microscope using a 20X oil immersion objective.

Marker gene amplification and 16S rRNA amplicon sequencing

All amplification protocols and cycling conditions used in this manuscript are provided in Table S4.

SI Results

Microbial communities in the egg masses are distinct from those in other seagrass microhabitats

Statistical differences in microbial community composition are displayed in Table S5 and alpha diversity between sample types is summarized in Table S6.

SI Discussion

The egg mass communities were highly distinct from sediment, seawater, and seagrass communities (Figure 6, Table S5), which resulted in 51 taxa having a significant association with the egg mass habitat (point biserial correlation, adjusted p-value <0.05). The numerically most abundant group in all egg masses was the class Alphaproteobacteria (rel. abundance of 39.4%) which is generally known to be among the most dominant groups in marine environments world-wide especially in the pelagic (1, 2, 3). Marine sediments are usually dominated by Gammaproteobacteria (4). Several studies consistently report Alpha- and Gammaproteobacteria as the most abundant members of oyster gut and gill microbial communities (5, 6, 7). Moreover, (8) noted that Alpha- and Gammaproteobacteria dominated the post-larvae stage of three oyster species (41-53% of the communities).

The most abundant member of the Alphaproteobacteria in all egg masses was the genus *Oceanicaulis* (2.68%) which belongs to the family *Hyphomonadaceae*, a group of motile or stalked, chemoheterotrophic bacteria isolated from seawater (9), reef-building corals (10) and dinoflagellate cultures (11). Their ability to adhere to surfaces using their prosthecae allows them to extend their cell surface area and thus take up nutrients more efficiently (12). Whether this could be an advantageous strategy to

thrive inside gelatinous egg masses of marine invertebrates and thus scavenge otherwise unavailable nutrients is up to debate. The second most abundant alphaproteobacterial member of all egg mass communities was *Lentilitoribacter donghaensis* which has previously been isolated from coastal seawaters of South Korea (13). Another well represented group of Alphaproteobacteria in the reproductive stages was the genus *Tropicimonas* which is a group of obligately halophilic bacteria isolated from seawater (14, 15) and marine sediments of a cage-cultured ark clam farm (16).

Figure S1: 16S rRNA gene tree of lucinid symbionts.

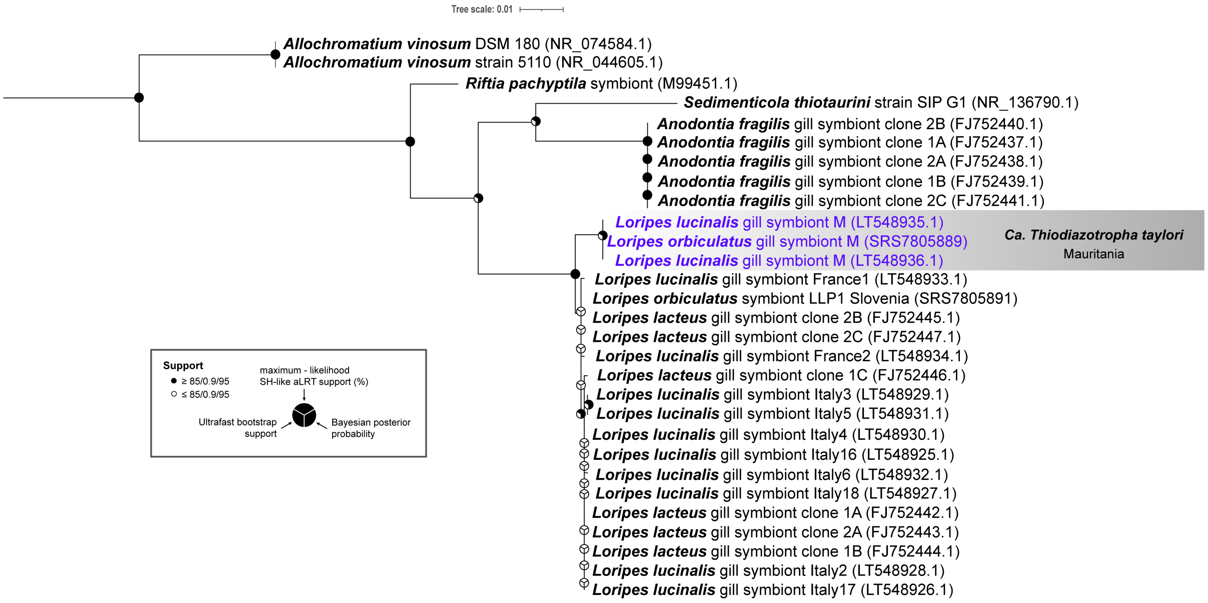

Figure S1. 16S rRNA gene tree of lucinid symbionts. Phylogenetic reconstruction of lucinid endosymbionts based on near full-length 16S rRNA gene sequences. Tree is rooted on *Allochromatium vinosum*. The substitution model K2P+I+G4 was automatically chosen by IQtree. Symbionts obtained from Mauritanian clams are highlighted in purple.

Figure S2: Localization of symbionts in the gill tissue of an adult bivalve and absence of these in the gills of larvae.

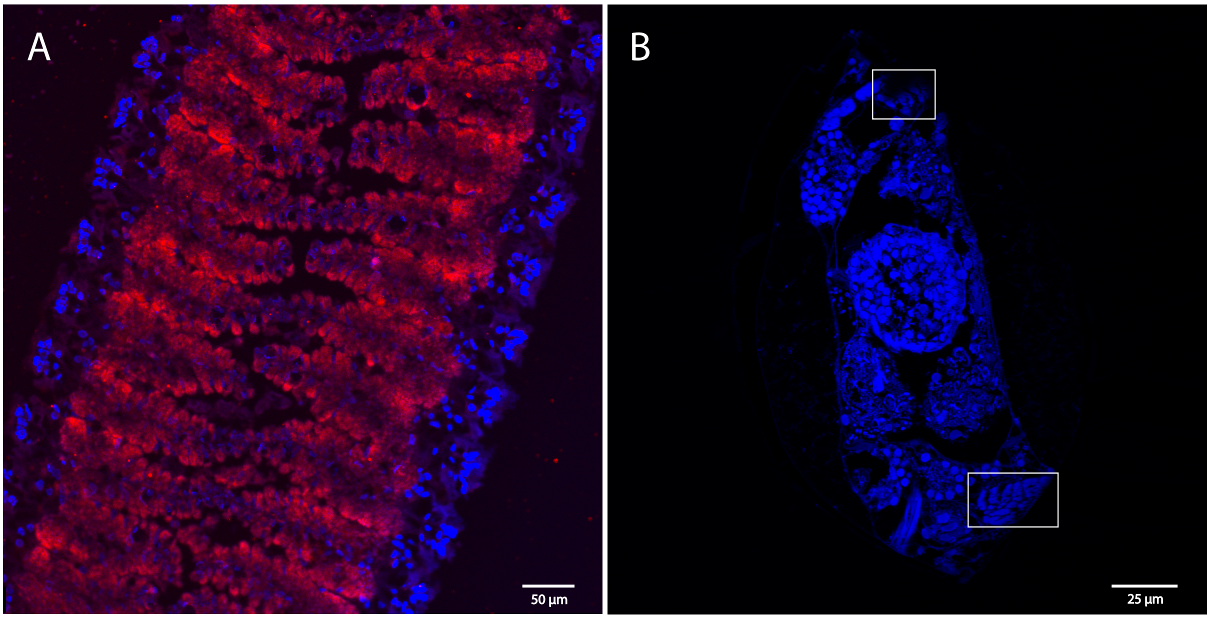

Figure S2. Distribution of endosymbionts in the gills of adult *L. orbiculatus* and absence of these in a 54-days-old larva from Mauritania. A) Cross section through a gill of an adult bivalve with the bacteriocyte-bearing zone depicted in red (symbiont-specific probe) and the ciliated, symbiont-free zone in blue. B) Cross-sectioned whole larva with two individual gills (highlighted by white frames) free of symbionts. Red, *Ca.* Thiodiazotropha taylori; blue, DAPI-labeled host nuclei.

Table S2. Primers and FISH probes used in this manuscript.

Probe/primer	Specificity	Sequence (5' - 3')	FA (%)*	Dye	Reference no.
symb845	*Ca.* T. endoloripes	TTAGCTGCGCCACTAAACCCT	35	Cy3	29
EUB338 I-III mix	Bacteria	EUB338 I: GCTGCCTCCCGTAGGTGT	35	ATTO488	27
	Bacteria	EUB338 II: GCAGCCACCCGTAGGTGT	35	ATTO488	28
	Bacteria	EUB338 III: GCTGCCACCCGTAGGTGT	35	ATTO488	28
nonEUB338	Bacteria	ACTCCTACGGGAGGCAGC	35	Fluos	27
27F	Bacteria	AGAGTTTGATCCTGGCTCAG	-	-	32, 33
1492R	Bacteria	GGYTACCTTGTTACGACTT	-	-	32, 33
cytb-F	Molluscs	GRGGKGCTACKGTAATTACTAA	-	-	30
cytbR_new	Molluscs	AAATAYCACTCGGGCTGGATATG	-	-	31
341F	Bacteria	CCT ACG GGN GGC WGC AG	-	-	35
785R	Bacteria	GAC TAC HVG GGT ATC TAA TCC	-	-	35

Table S3: FISH reagents used in this study.

Reagent	Hybridization Buffer (6ml) 35% FA*	Washing Buffer (50ml) 35% FA*
5M NaCl	1.08	0.7
1M Tris/HCl	0.12	1
MilliQ	2.7	47.8
Formamide	2.1	-
10% SDS	6 µl	50 µl
0.5M EDTA	-	0.5

*FA = Formamide concentration used

Table S5. PERMANOVA results showing differences in microbial community composition among microhabitat types (a) and differences in composition between egg masses with and without larvae (b).

a. Among Microhabitat Types (Egg Mass, Leaf, Root, Seawater, and Sediment)

PERMANOVA
Formula = Microbial Community Composition ~ Microhabitat Type

	DF	Sum of Sqs	R2	F	p-value
Treatment Time	4	12.866	0.25244	9.202	0.001
Residual	109	38.099	0.74756		
Total	113	50.965	1		

b. Between Egg Masses with and without Larvae

PERMANOVA
Formula = Microbial Community Composition ~ Presence of Larvae

	DF	Sum of Sqs	R2	F	p-value
Treatment Time	1	0.2032	0.01996	0.6313	0.881
Residual	31	9.9786	0.98004		
Total	32	10.1818	1		

Table S6. Alpha diversity by microhabitat type. Alpha diversity metrics (Shannon-Weiner Index, Pielou's Evenness Index, and number of taxonomic groups observed) for
microbial communities from the egg masses and seagrass environment (5 sample types). Values are medians followed by the range in parentheses.

Microhabitat Type	Diversity (*H'*)	Evenness (*J*)	Taxonomic Groups Observed
Egg Masses	2.98 (1.56-4.18)	0.90 (0.75-0.94)	27 (8-111)
Seagrass Leaf	2.59 (1.74-3.35)	0.82 (0.73-0.89)	24 (7-49)
Seagrass Root	3.88 (2.12-4.59)	0.86 (0.78-0.96)	77 (14-184)
Seawater	3.47 (2.80-3.65)	0.88 (0.79-0.93)	49 (23-59)
Sediment	4.24 (3.77-4.50)	0.86 (0.78-0.90)	126.5 (93-176)

SI References:

1. Morris RM, Rappé MS, Connon SA, Vergin KL, Siebold WA, Carlson CA, *et al.* SAR11 clade dominates ocean surface bacterioplankton communities. Nature 2002; 420:806-810.

2. Kersters K, Vos P, Gillis M, Swings J, Vandamme P, Stackenbrandt E. Introduction to the Proteobacteria. Prokaryotes 2006; 5: 3–37.

3. Schiaffino MR, Sánchez ML, Gerea M, Unrein F, Balagué V, Gasol JM, *et al.* Distribution patterns of the abundance of major bacterial and archaeal groups in Patagonian lakes. J Plankton Res 2016; 38:64-82.

4. Wang Y, Sheng HF, He Y, Wu JY, Jiang YX, Tam NFY, *et al.* Comparison of the levels of bacterial diversity in freshwater, intertidal wetland, and marine sediments by using millions of illumina tags. Appl Environ Microbiol 2012; 78:8264-8271.

5. Pujalte MJ, Ortigosa M, Macián MC, Garay E. Aerobic and facultative anaerobic heterotrophic bacteria associated to Mediterranean oysters and seawater. Int Microbiol 1999; 2:259-266.

6. Green TJ, Barnes AC. Bacterial diversity of the digestive gland of Sydney rock oysters, *Saccostrea glomerata* infected with the paramyxean parasite, *Marteilia sydneyi.* J Appl Microbiol 2010; 109:613-622.

7. Wegner KA, Volkenborn N, Peter H, Eiler A. Disturbance induced decoupling between host genetics and composition of the associated microbiome. BMC Microbiol 2013; 13:252.

8. Trabal Fernández N, Mazón-Suástegui JM, Vázquez-Juárez R, Ascencio-Valle F, Romero J. Changes in the composition and diversity of the bacterial microbiota associated with oysters (*Crassostrea corteziensis*, *Crassostrea gigas* and

Crassostrea sikamea) during commercial production. FEMS Microbiol Ecol, 2014; 88:69-83.

9. Moore RL, Weiner RM, Gebers R. Genus *Hyphomonas* Pongratz 1957 nom. rev. emend., *Hyphomonas polymorpha* Pongratz 1957 nom. rev. emend., and *Hyphomonas neptunium* (Leifson 1964) comb. nov. emend. (*Hyphomicrobium neptunium*). Int J Syst Evol Micr 1984; 34:71-73.
10. Chen MH, Sheu SY, Chen CA, Wang JT, Chen WM. *Oceanicaulis stylophorae* sp. nov., isolated from the reef-building coral *Stylophora pistillata*. Int J Syst Evol Micr 2012; 62:2241-2246.
11. Strömpl C, Hold GL, Lünsdorf H, Graham J, Gallacher S, Abraham WR, *et al.* *Oceanicaulis alexandrii* gen. nov., sp. nov., a novel stalked bacterium isolated from a culture of the dinoflagellate *Alexandrium tamarense* (Lebour) Balech. Int J Syst Evol Micr 2003; 53:1901-1906.
12. McAdams HH. Bacterial stalks are nutrient-scavenging antennas. Proc Natl Acad Sci USA 2006; 103:1435-11436.
13. Park S, Lee JS, Lee KC, Yoon JH. *Lentilitoribacter donghaensis* gen. nov., sp. nov., a slowly-growing alphaproteobacterium isolated from coastal seawater. Antonie van Leeuwenhoek, 2013; 103:457-464.
14. Harwati TU, Kasai Y, Kodama Y, Susilaningsih D, Watanabe K. *Tropicimonas isoalkanivorans* gen. nov., sp. nov., a branched-alkane-degrading bacterium isolated from Semarang Port in Indonesia. Int J Syst Evol Micr 2009; 59:388-391.
15. Oh KH, Choi WC, Jung YT, Kang SJ, Oh TK, Yoon JH. *Tropicimonas aquimaris* sp. nov., isolated from seawater, and emended description of the genus *Tropicimonas* Harwati et al. 2009. Int J Syst Evol Micr, 2012; 62:688-692.

16. Shin NR, Roh SW, Kim MS, Yun B, Whon TW, Kim YO, *et al. Tropicimonas sediminicola* sp. nov., isolated from marine sediment. Int J Syst Evol Micr 2012; 62:2424-2429
