# Supplementary material for: Microbial communities in developmental stages of lucinid bivalves
Source: ISME Commun. 2022 Jul 8;2:56. doi: 10.1038/s43705-022-00133-4 (PMC9723593; doi:10.1038/s43705-022-00133-4)
Supplement: Supplementary file 2 — Table S1 [file 43705_2022_133_MOESM2_ESM.pdf]

Table S1. List of samples used for the ordination of ASV abundance.

Sample_Specific	Host Species	Study_Site	Sample_Date	Without Larvae	Original Name	# NCBI BioProject
Egg_Mass	Loripes_lucinalis	Mauritania	Dec_2015	No	SAMPLE.539.ESM.2.F.1.filtered.fastq.gz	
Egg_Mass	Loripes_lucinalis	Mauritania	Dec_2015	No	SAMPLE.540.ESM.3.F.1.filtered.fastq.gz	
Egg_Mass	Loripes_lucinalis	Mauritania	Dec_2015	No	SAMPLE.541.ESM.4.F.1.filtered.fastq.gz	
Egg_Mass	Loripes_lucinalis	Mauritania	Dec_2015	No	SAMPLE.542.ESM.5.F.1.filtered.fastq.gz	
Egg_Mass	Loripes_lucinalis	Mauritania	Dec_2015	No	SAMPLE.543.ESM.6.F.1.filtered.fastq.gz	
Egg_Mass	Loripes_lucinalis	Mauritania	Dec_2015	No	SAMPLE.544.ESM.7.F.1.filtered.fastq.gz	
Egg_Mass	Loripes_lucinalis	Mauritania	Dec_2015	No	SAMPLE.545.ESM.9.F.1.filtered.fastq.gz	
Egg_Mass	Loripes_lucinalis	Mauritania	Dec_2015	No	SAMPLE.538.ESM.10.F.1.filtered.fastq.gz	
Egg_Mass	Loripes_lucinalis	Mauritania	Dec_2015	No	SAMPLE.211.DecEgg.sac.4.F.1.filtered.fastq.gz.gz	
Egg_Mass	Loripes_lucinalis	Mauritania	Dec_2015	No	SAMPLE.213.DecEgg.sac.5.F.1.filtered.fastq.gz.gz	
Egg_Mass	Loripes_lucinalis	Mauritania	Dec_2015	No	SAMPLE.215.DecEgg.sac.7.F.1.filtered.fastq.gz.gz	
Egg_Mass	Loripes_lucinalis	Mauritania	Dec_2015	No	SAMPLE.259.Decegg.sac.6.F.1.filtered.fastq.gz.gz	
Egg_Mass	Loripes_lucinalis	Mauritania	Dec_2015	No	SAMPLE.217.DecEgg.sac.8.F.1.filtered.fastq.gz.gz	
Egg_Mass	Loripes_lucinalis	Mauritania	Dec_2015	No	SAMPLE.219.DecEgg.sac.9.F.1.filtered.fastq.gz.gz	
Egg_Mass	Loripes_lucinalis	Mauritania	Dec_2015	No	SAMPLE.205.DecEgg.sac.14.F.1.filtered.fastq.gz.gz	
Egg_Mass	Loripes_lucinalis	Mauritania	Dec_2015	No	SAMPLE.207.DecEgg.sac.15.F.1.filtered.fastq.gz.gz	
Egg_Mass	Loripes_lucinalis	Mauritania	Dec_2015	No	SAMPLE.209.DecEgg.sac.16.F.1.filtered.fastq.gz.gz	
Egg_Mass	Loripes_lucinalis	Mauritania	Dec_2015	Yes	SAMPLE.546.ESM.11.F.1.filtered.fastq.gz	
Egg_Mass	Loripes_lucinalis	Mauritania	Dec_2015	Yes	SAMPLE.547.ESM.13.F.1.filtered.fastq.gz	
Egg_Mass	Loripes_lucinalis	Mauritania	Dec_2015	Yes	SAMPLE.221.control.DecEgg.mass.3.F.1.filtered.fastq.gz.gz	
Egg_Mass	Loripes_lucinalis	Mauritania	Dec_2015	Yes	SAMPLE.225.control.DecEgg.mass.5.F.1.filtered.fastq.gz.gz	
Egg_Mass	Loripes_lucinalis	Mauritania	Jan_2017	No	SAMPLE.239.JanEgg.sac.5.F.1.filtered.fastq.gz.gz	
Egg_Mass	Loripes_lucinalis	Mauritania	Jan_2017	No	SAMPLE.241.JanEgg.sac.6.F.1.filtered.fastq.gz.gz	
Egg_Mass	Loripes_lucinalis	Mauritania	Jan_2017	No	SAMPLE.243.JanEgg.sac.10.F.1.filtered.fastq.gz.gz	
Egg_Mass	Loripes_lucinalis	Mauritania	Jan_2017	No	SAMPLE.227.JanEgg.sac.17.F.1.filtered.fastq.gz.gz	
Egg_Mass	Loripes_lucinalis	Mauritania	Jan_2017	No	SAMPLE.229.JanEgg.sac.18.F.1.filtered.fastq.gz.gz	
Egg_Mass	Loripes_lucinalis	Mauritania	Jan_2017	No	SAMPLE.231.JanEgg.sac.19.F.1.filtered.fastq.gz.gz	
Egg_Mass	Loripes_lucinalis	Mauritania	Jan_2017	No	SAMPLE.233.JanEgg.sac.23.F.1.filtered.fastq.gz.gz	
Egg_Mass	Loripes_lucinalis	Mauritania	Jan_2017	No	SAMPLE.235.JanEgg.sac.24.F.1.filtered.fastq.gz.gz	
Egg_Mass	Loripes_lucinalis	Mauritania	Jan_2017	No	SAMPLE.237.JanEgg.sac.25.F.1.filtered.fastq.gz.gz	
Egg_Mass	Loripes_lucinalis	Mauritania	Jan_2017	Yes	SAMPLE.245.control.JanEgg.mass.5.F.1.filtered.fastq.gz.gz	

Egg_Mass	Loripes_lucinalis	Mauritania	Jan_2017	Yes	SAMPLE.247.control.JanEgg.mass.9.F.1.filtered.fastq.gz.gz	
Egg_Mass	Loripes_lucinalis	Mauritania	Jan_2017	Yes	SAMPLE.249.control.JanEgg.mass.6.F.1.filtered.fastq.gz.gz	
Surface		Mauritania	2015		Ma_1A__A.F.1.filtered.fastq.gz.gz	
Intermediate		Mauritania	2015		Ma_1B__A.F.1.filtered.fastq.gz.gz	
Bottom		Mauritania	2015		Ma_1C__A.F.1.filtered.fastq.gz.gz	
Rhizome_Debris		Mauritania	2015		Ma_3__A.F.1.filtered.fastq.gz.gz	
Rhizome_Debris		Mauritania	2015		Ma_4__A.F.1.filtered.fastq.gz.gz	
Root	Zostera_marina	NE_Atlantic	Jun_2013		SRR1994759	PRJNA282077
Root	Zostera_marina	NE_Atlantic	Jun_2013		SRR1994760	PRJNA282077
Root	Zostera_marina	NE_Atlantic	Jun_2013		SRR1994761	PRJNA282077
Root	Zostera_marina	NE_Atlantic	Jun_2013		SRR1994763	PRJNA282077
Root	Zostera_noltii	NE_Atlantic	Jun_2013		SRR1994764	PRJNA282077
Root	Zostera_noltii	NE_Atlantic	Jun_2013		SRR1994765	PRJNA282077
Root	Zostera_noltii	NE_Atlantic	Jun_2013		SRR1994767	PRJNA282077
Root	Zostera_noltii	NE_Atlantic	Jun_2013		SRR1994768	PRJNA282077
Root	Zostera_noltii	NE_Atlantic	Jun_2013		SRR1994769	PRJNA282077
Root	Cymodocea_nodc	NE_Atlantic	Jun_2013		SRR1994770	PRJNA282077
Root	Cymodocea_nodc	NE_Atlantic	Jun_2013		SRR1994771	PRJNA282077
Root	Cymodocea_nodc	NE_Atlantic	Jun_2013		SRR1994772	PRJNA282077
Root	Cymodocea_nodc	NE_Atlantic	Jun_2013		SRR1994773	PRJNA282077
Root	Cymodocea_nodc	NE_Atlantic	Jun_2013		SRR1994774	PRJNA282077
Sediment		NE_Atlantic	Jun_2013		SRR2000140	PRJNA282077
Sediment		NE_Atlantic	Jun_2013		SRR2000141	PRJNA282077
Sediment		NE_Atlantic	Jun_2013		SRR2000142	PRJNA282077
Sediment		NE_Atlantic	Jun_2013		SRR2000143	PRJNA282077
Sediment		NE_Atlantic	Jun_2013		SRR2000144	PRJNA282077
Coastal_Lagoon		NE_Atlantic	Jun_2013		SRR2000145	PRJNA282077
Coastal_Lagoon		NE_Atlantic	Jun_2013		SRR2000158	PRJNA282077
Coastal_Lagoon		NE_Atlantic	Jun_2013		SRR2000159	PRJNA282077
Coastal_Lagoon		NE_Atlantic	Jun_2013		SRR2000192	PRJNA282077
Coastal_Lagoon		NE_Atlantic	Jun_2013		SRR2000193	PRJNA282077
Root	Zostera_marina	NE_Atlantic	Sep_2013		SRR2000194	PRJNA282077
Root	Zostera_marina	NE_Atlantic	Sep_2013		SRR2000195	PRJNA282077
Root	Zostera_marina	NE_Atlantic	Sep_2013		SRR2000197	PRJNA282077

Root	Zostera_marina	NE_Atlantic	Sep_2013	SRR2000198	PRJNA282077
Root	Zostera_marina	NE_Atlantic	Sep_2013	SRR2000199	PRJNA282077
Root	Zostera_noltii	NE_Atlantic	Sep_2013	SRR2000200	PRJNA282077
Root	Zostera_noltii	NE_Atlantic	Sep_2013	SRR2000201	PRJNA282077
Root	Zostera_noltii	NE_Atlantic	Sep_2013	SRR2000202	PRJNA282077
Root	Zostera_noltii	NE_Atlantic	Sep_2013	SRR2000203	PRJNA282077
Root	Zostera_noltii	NE_Atlantic	Sep_2013	SRR2000204	PRJNA282077
Sediment		NE_Atlantic	Sep_2013	SRR2000205	PRJNA282077
Sediment		NE_Atlantic	Sep_2013	SRR2000210	PRJNA282077
Sediment		NE_Atlantic	Sep_2013	SRR2000212	PRJNA282077
Sediment		NE_Atlantic	Sep_2013	SRR2000213	PRJNA282077
Sediment		NE_Atlantic	Sep_2013	SRR2000214	PRJNA282077
Sediment		NE_Atlantic	Sep_2013	SRR2000215	PRJNA282077
Sediment		NE_Atlantic	Sep_2013	SRR2000217	PRJNA282077
Sediment		NE_Atlantic	Sep_2013	SRR2000235	PRJNA282077
Sediment		NE_Atlantic	Sep_2013	SRR2000237	PRJNA282077
Sediment		NE_Atlantic	Sep_2013	SRR2000238	PRJNA282077
Leaf	Zostera_japonica	Oregon	Sep_2014	SRR6308580	PRJNA41903
Leaf	Zostera_japonica	Oregon	Sep_2014	SRR6308581	PRJNA41903
Leaf	Zostera_marina	Oregon	Sep_2014	SRR6308582	PRJNA41903
Root	Zostera_japonica	Oregon	Sep_2014	SRR6308584	PRJNA41903
Root	Zostera_japonica	Oregon	Sep_2014	SRR6308585	PRJNA41903
Root	Zostera_marina	Oregon	Sep_2014	SRR6308587	PRJNA41903
Leaf	Zostera_marina	Oregon	Sep_2014	SRR6308588	PRJNA41903
Leaf	Zostera_marina	Oregon	Sep_2014	SRR6308589	PRJNA41903
Leaf	Zostera_japonica	Oregon	Sep_2014	SRR6308590	PRJNA41903
Root	Zostera_japonica	Oregon	Sep_2014	SRR6308591	PRJNA41903
Leaf	Zostera_marina	Oregon	Sep_2014	SRR6308592	PRJNA41903
Root	Zostera_marina	Oregon	Sep_2014	SRR6308593	PRJNA41903
Brackish		Oregon	Jul_2014	SRR6308594	PRJNA41903
Brackish		Oregon	Jul_2014	SRR6308595	PRJNA41903
Leaf	Zostera_marina	Oregon	Jul_2014	SRR6308596	PRJNA41903
Root	Zostera_marina	Oregon	Jul_2014	SRR6308597	PRJNA41903
Leaf	Zostera_marina	Oregon	Jul_2014	SRR6308598	PRJNA41903

Root	Zostera_marina	Oregon	Jul_2014	SRR6308599	PRJNA41903
Leaf	Zostera_japonica	Oregon	Jul_2014	SRR6308600	PRJNA41903
Root	Zostera_japonica	Oregon	Jul_2014	SRR6308601	PRJNA41903
Root	Zostera_marina	Oregon	Sep_2014	SRR6308602	PRJNA41903
Root	Zostera_marina	Oregon	Sep_2014	SRR6308603	PRJNA41903
Leaf	Zostera_japonica	Oregon	Sep_2014	SRR6308604	PRJNA41903
Leaf	Zostera_japonica	Oregon	Sep_2014	SRR6308605	PRJNA41903
Seawater		Oregon	Sep_2014	SRR6308606	PRJNA41903
Seawater		Oregon	Sep_2014	SRR6308607	PRJNA41903
Root	Zostera_japonica	Oregon	Sep_2014	SRR6308608	PRJNA41903
Root	Zostera_japonica	Oregon	Sep_2014	SRR6308609	PRJNA41903
Root	Zostera_japonica	Oregon	Jul_2014	SRR6308610	PRJNA41903
Seawater		Oregon	Sep_2014	SRR6308611	PRJNA41903
Seawater		Oregon	Sep_2014	SRR6308612	PRJNA41903
Leaf	Zostera_japonica	Oregon	Jul_2014	SRR6308613	PRJNA41903
