# Supplementary material for: Microbial communities in developmental stages of lucinid bivalves
Source: ISME Commun. 2022 Jul 8;2:56. doi: 10.1038/s43705-022-00133-4 (PMC9723593; doi:10.1038/s43705-022-00133-4)
Supplement: Supplementary file 3 — Table S4 [file 43705_2022_133_MOESM3_ESM.pdf]

Table S4. Amplification protocols and cycling conditions used for Sanger and MiSeq Amplicon Sequencing.

	Reagents per reaction	Time (min)
16S PCR	17.525 µl ddH2O	1x 94°C for 04:00
(25 µl reaction)	2.5 µl 10X DreamTaq Buffer	32x 94°C for 00:30
	2.5 µl dNTP mix (2 mM)	32x 52°C for 00:45
	0.625 µl 27F Primer (10 pmol/µl)	32x 72°C for 00:45
	0.625 µl 1492R Primer (10 pmol/µl)	1x 72°C for 10:00
	0.1 µl BSA (20mg/ml)	
	0.125 µl DeamTaq Polymerase (5U/ µl)	
	1 µl purified DNA template	
cytB PCR	15.275 µl ddH2O	1x 95°C for 05:00
(25 µl reaction)	2.5 µl 10X DreamTaq Buffer	32x 95°C for 01:00
	2.5 µl dNTP mix (2 mM)	32x 45°C for 01:00
	0.250 µl cytb_F Primer (10 pmol/µl)	32x 72°C for 01:30
	0.250 µl cytb_R_new Primer (10 pmol/µl	1x 72°C for 10:00
	0.1 µl BSA (20mg/ml)	
	3 µl MgCl2 (25mg/ml)	
	0.125 µl DeamTaq Polymerase (5U/ µl)	
	1 µl purified DNA template	
1st step PCR	17.525 µl ddH$_2$O	1x 94°C for 04:00
egg masses	2.5 µl DreamTaq Green buffer (10x)	25x 94°C for 00:30
(25 µl reaction)	2.5 µl dNTP mix (2 mM)	25x 52°C for 00:45
	0.1 µl BSA (20 µg/µl)	25x 72°C for 00:45
	0.625 µl Primer 341 F (10 µM)	1x 72°C for 10:00
	0.625 µl Primer 785 R (10 µM)	
	0.125 µl Dream Taq Polymerase (5U/µl)	
	1 µl purified DNA template	
1st step PCR	12.4 µl ddH$_2$O	1x 95°C for 05:00
sediment, seagrass	2 µl DreamTaq Green buffer (10x)	25x 95°C for 00:30
(20 µl reaction)	2 µl dNTP mix (2 mM)	25x 50°C for 00:30
	0.1 µl BSA (20 µg/µl)	25x 72°C for 01:00
	1.6 µl MgCl2 (25mM)	1x 72°C for 10:00
	0.4 µl Primer 341 F (50 µM)	
	0.4 µl Primer 785 R (50 µM)	
	0.1 µl Dream Taq Polymerase (5U/µl)	
	1 µl purified DNA template	
2nd step Barcode PCR	35.75 µl ddH$_2$O	1x 94°C for 04:00
egg masses	5 µl DreamTaq Green buffer (10x)	10x 94°C for 00:30
(50 µl reaction)	5 µl dNTP mix (2mM)	10x 52°C for 00:45
	0.2 µl BSA (20 µg/µl)	10x 72°C for 00:45
	0.8 µl Barcode (50 µM)	1x 72°C for 10:00
	0.25 µl DreamTaq Polymerase (5U/µl)	

	3 µl purified template DNA	
2nd step Barcode PCR sediment, seagrass (20 µl reaction)	12.4 µl ddH_2O<br>2 µl DreamTaq Green buffer (10x)<br>2 µl dNTP mix (2mM)<br>0.1 µl BSA (20 µg/µl)<br>1.6 µl MgCl2 (25mM)<br>0.8 µl Barcode (50 µM)<br>0.1 µl DreamTaq Polymerase (5U/µl)<br>1 µl purified template DNA	1x 95°C for 05:00<br>5x 95°C for 00:30<br>5x 52°C for 00:30<br>5x 72°C for 01:00<br>1x 72°C for 10:00
